# A comparison of perceptual-cognitive skills in expert and non-expert sports officials: a systematic review and meta-analysis

**DOI:** 10.3389/fpsyg.2024.1380281

**Published:** 2024-06-21

**Authors:** Yidong Wu, Ziqi Yang, Rishu Wang, Hongtao Zeng, Qi Zhang

**Affiliations:** ^1^School of Economics and Management, Shanghai University of Sport, Shanghai, China; ^2^School of Athletic Performance, Shanghai University of Sport, Shanghai, China; ^3^School of Physical Education, Huazhong University of Science and Technology, Wuhan, China

**Keywords:** perceptual-cognitive skills, visual search, sports officials, systematic review, meta-analysis

## Abstract

**Introduction:**

The purpose of this study is to systematically compare and assess the differences in perceptual-cognitive skills between expert and non-expert sports officials, and further explore the potential differences caused by different types of sports officials, in order to provide a more comprehensive understanding of the perceptual-cognitive skills of sports officials.

**Methods:**

Relevant literature published before 31 December 2022 was searched in four English databases. Review Manager 5.4 and Stata 12.0 software were used for meta-analysis and bias test.

**Results:**

Expert sports officials are significantly more accurate in their decision-making than non-expert sports officials, and exhibit a large amount of effect size (SMD = 1.09; 95%CI: 0.52, 1.66; *P* < 0.05). Expert sports officials had significantly fewer number of fixations than non-expert sports officials and showed a moderate amount of effect size (SMD = 0.71; 95%CI: 1.25, 0.17; *P* < 0.05). Expert sports officials' duration of fixation (SMD = 0.23; 95%CI: 0.25, 0.71; *P* = 0.35) were not significantly different from non-expert sports officials.

**Discussion:**

It can be seen that there are differences in the Perceptual-cognitive skills of expert and non-expert sports officials. Decision-making accuracy can serve as an important indicator for distinguishing the perceptual-cognitive skills of expert and non-expert sports officials. Number of fixations can serve as important indicators to differentiate the perceptual-cognitive skills of monitors.

**Systematic review registration:**

https://www.crd.york.ac.uk/PROSPERO/display_record.php?RecordID=418594, identifier: CRD42023418594.

## 1 Introduction

In the process of movement, the environment often contains a large amount of information. However, human capacity to process these information is limited, and active observer has to select the relevant signs from the large amount of information available in the environment (Moeinirad et al., 2020). This ability of an individual to identify and acquire environmental information and integrate it with existing knowledge in order to process information and execute appropriate responses in complex tasks is known as perceptual-cognitive skills (Marteniuk, 1976). Related research has demonstrated that the ability to acquire visual information and to select and execute appropriate movements is key to high-level performance and even plays a critical role in sports (Williams and Ericsson, 2005; Williams et al., 2011). Skilled experts have been found that to be superior on a variety of perceptual-cognitive tasks. Such as anticipation, the ability to predict the action of others based on early visual information (Abernethy and Russell, 1987); Decision-making, the ability to select the best option from a variety of alternatives (Helsen and Pauwels, 1993). Recall, the ability to recall previously seen situations (Allard et al., 1980). It has been shown that experts tend to be able to focus their attention on searching for relevant cues and making quick and accurate decisions in a game situation filled with a lot of information, while non-experts are less able to do so. Thus, it is these perceptual-cognitive skills that often serve as an important basis to distinguish experts from non-experts (Ward and Williams, 2003; Mann et al., 2007).

A large number of researchers have made it clear that with certain visual search behavior expert athletes can make proficient anticipation, alleviate the temporal constraints of the task and make fast and more accurate decisions than non-expert athletes (Helsen and Starkes, 1999). The investigation of gaze behavior seems to be an appropriate approach to better understand the visual attention and the perceptual-cognitive processes in information search (Mack, 2020). Williams and Ericsson (2005) argue that with certain visual search strategies, experts can achieve anticipation of extended movement cues, recognition of movement pattern, and the use of information. In sports situations, athletes need to pay attention to the most critical cues if they want to achieve superior performance. Therefore, more research has attempted to study the perceptual-cognitive skills of athletes in terms of selective attention and visual search abilities. The expert-novice research paradigm is currently the main research paradigm in the field of eye movements and provides an important basis for exploring experts high-level motor performance. Some researchers have shown that gaze behavior can be explicitly used as a process tracing measure for decision making. The decision making skills of expert athletes could also be directly traced to gaze behavior, thereby making the study of gaze behavior essential for all sports (Mann et al., 2009).

Indeed, numerous researchers have used eye tracking systems to test the gaze patterns of athletes as they attempt to anticipate or judge skilled performance in both laboratory and field settings (Mann et al., 2019). Researchers often focus on the location, sequence, number, and duration of fixations in athletes. The location and sequence of fixations are important enough to reflect the signs used in decision-making, and the number and duration of fixations reflect the need for information processing and the allocation of attention. There have also been some experimental studies that have measured the performance of athletes when using perceptual-cognitive skills for decision-making through reaction time and response accuracy (Williams et al., 1994; Guizani et al., 2006; Piras et al., 2014; Ottoboni et al., 2015). Reaction time refers to the objective length of time between the onset of a stimulus and the production of an apparent response, while response accuracy is the frequency of producing in which an appropriate response is made according to objective standards and task constraints. In general, expert athletes can demonstrate better accuracy over short period of time after a long period of training. They spend less time scanning for relevant environmental information, which keeps their attention focused on important place and lasts longer. Vickers and Adolphe (1997) used eye movement techniques to study the visual tracking of expert and non-expert volleyball players and found that expert players tracked the ball earlier and visually tracked the ball longer than non-experts. Panchuk and Vickers (2006) in their study of visual gaze characteristics of ice hockey goalies, found that ice hockey goalies' accurate spatial and temporal judgment of incoming pucks was dependent on the timing of visual orientation and visual tracking prior to the save. Great hockey goalies look at the incoming puck sooner, keep their eyes on the puck for longer before making a save, and rarely look at the attacking player's body. Thus, longer fixation times seem to imply more extraction of important information as well as more detailed information processing. The fundamental difference between experts and non-experts seems to be that experts are better able to extract and process information distributed throughout the body (Abernethy et al., 2008).

While abundant research has shown how athletes make decisions at a perceptual-cognitive level, relatively little research has focused on the decision-making performance and perceptual-cognitive skills of sports officials. Given the association among anticipation, gaze behavior, and decision-making accuracy in athletes, it is important to study these perceptual-cognitive processes in sports officials (Mann et al., 2007). Sports officials have played an essential and significant role in today's sports events since the emergence of modern athletics. They are appointed in most sporting competitions to ensure that the rules of the game are implemented (Bar-Eli et al., 2011). The sports officials are crucial in maintaining the fairness and impartiality of sporting events, and their activities directly affects the athletes' abilities. Indeed, perceptual-cognitive skills are just as important for sports officials as for athletes. Sports officials required to observe a large amount of information under strict time constrains, and use these information to make timely and accurate decision which are heavily scrutinized by athletes, coaches and spectators (Plessner and Haar, 2006). The main task of a sports official in a game is to accurately perceive complex situations, quickly process key cues, and consistently make correct and reasonable decisions (MacMahon et al., 2014). Perceptual-cognitive superiority in the sports domain can be assessed either in a sport-specific context representing the requirements of a competitive and realistic setting (domain-specific skills, such as decision-making performance) or by use of more generic tests with no direct link to the performance setting (domain-generic skills, such as local information processing; Spitz et al., 2018). Expert sports officials tend to perform better than non-experts on domain-specific tests, but have more complex results on domain-specific skills. The reason for the difference in the performance of expert and non-expert sports officials may be related to the anticipation of sports officials. Expert sports officials can rely on spatial or time anticipation to make predictions about the environment and determine behavior (Schrödter et al., 2023). Therefore, it is pivotal to examine how sports officials direct their vision to obtain information from the game, and subsequently make decisions at the perceptual-cognitive level, for a comprehensive assessment of their performance.

As previously noted, sports officials' decisions and predictions are affected by visual search behavior at the perceptual-cognitive level, enabling them to execute their activities with greater efficiency. Current academic research on sports officials' perceptual-cognitive skills, visual search behavior, and decision-making is considerably less prevalent than that of athletes. Although the total number of related studies is limited and their findings are inconclusive, certain scholars have experimentally demonstrated differences among sports officials in perceptual-cognitive skills and visual search behavior. Aghakhanpour et al. (2021) examined the decision-making and visual search patterns of fencing referees, revealing that expert referees demonstrated greater decision-making accuracy than novice referees. Furthermore, expert referees had fewer fixations and longer fixation duration compared to novice referees. Similar findings were discovered in Bard et al. (1980) research on gymnastics judges and Kostrna and Tenenbaum (2022) research on baseball umpires. However, studies by Hancock and Ste-Marie (2013) and Catteeuw et al. (2009) point out that there is no difference between expert and non-expert referees in visual search behavior. Ziv et al. (2020) conducted a review of sports officials' visual behavior in different sports. Their analysis of 12 studies showed that seven studies revealed variations in visual behavior among sports officials of different skill levels, while the remaining four studies found no differences. The authors suggested that sports officials display distinctive visual search patterns across various sport contexts and tasks. In fact, the decision-making demands of sports officials will also vary depending on the complexity of the task. To help explain the differences in sporting officials' performance demands, MacMahon classified officials by their respective movement, perceptual and competition interaction demands. This resulted in three specific groups of sports officials including, monitors (such as gymnastics judge), reactors (such as tennis line judge), and interactors (such as soccer referee; MacMahon et al., 2014). In these three types of sports officials, interactor have greater movement and fitness demands and are required to process multiple decision cues and interact with greater numbers of players. For example, interactors' decisions are often made under strict time and information constraints, require deep prior knowledge and efficiency in appraising and processing perceptual information, and involve a high degree of mental and physical fatigue (Kittel et al., 2021). Such complex demands may make the interactors inferior to the monitors and reactors in perceptual-cognitive performance. Although sports officials can be classified as interactors, monitors and reactors, further research is needed to clarify the specific differences in perceptual-cognitive skills between expert and non-expert sports officials.

Based on the above theories and background, the purpose of this study is to systematically compare and assess the differences in perceptual-cognitive skills between expert and non-expert sports officials, and further explore the potential differences caused by different types of sports officials, in order to provide a more comprehensive understanding of the perceptual-cognitive skills of sports officials. We applied meta-analysis to help us quantitatively evaluate specific differences in perceptual-cognitive skills between expert and non-expert sports officials. In addition, different types of sports officials face different task demands, which can lead to different criteria of decision-making and visual search strategies. Given that the type of sports official may be a potential factor affecting the perceptual-cognitive skills of experts and non-experts, we divided sports officials into interactors, reactors and monitors to further analyze and discuss according to MacMahon's classification.

## 2 Methods

### 2.1 Search strategies

The study protocol for this systematic review and meta-analysis was registered at PROSPERO with the registration number (CRD42023418594). To search as much relevant articles as possible, a systematic and comprehensive search was conducted in four English databases: Pubmed, Web of Science, EBSCO-SPORTDiscus, and EBSCO-MEDLINE. Specific search criteria were established following the PRISMA guidelines, the preferred report for systematic review and meta-analysis. Two independent researchers performed an initial screening of the titles and abstracts. Relevant articles published prior to 31st December 2022 was primarily searched. In each database, the keywords used when searching were as followed: (anticipation OR prediction OR decision-making OR expertise OR cue use OR information processing OR cognitive characteristics OR visual search OR visual attention OR visual fixation OR eye movement OR eye-tracking OR perceptual cognitive skills OR expert OR non-expert OR amateur OR novice OR elite) AND (referee OR judge OR judgment OR umpire OR official OR officiate). The detailed search process can be found in the Supplementary Table 1.

### 2.2 Inclusion and exclusion criteria

The study included the articles according to the following criteria: (1) Full-text articles. (2) Article published in English. (3) Articles must be based on an expert/non-expert paradigm and include both expert and non-expert groups. (4) Articles should report the sample size of the participants as well as the mean and standard deviation of the relevant metrics. In addition, the inclusion criteria of selected studies were based on the PICOS principle: P-participant, sports officials. I-intervention, experts with more expertise and experience. C-comparison, non-experts have less expertise and experience. O-outcome, results related to perceptual-cognitive skills include decision-making accuracy, number of fixation, and duration of fixation. S-study, study with expert/non-expert research paradigm.

Articles that does not meet the following criteria will also be excluded: (1) Means and standard errors for variables in the study are not reported. (2) Abstracts, research programme, news reports, dissertations, reviews and case reports from congress meetings or proceedings. (3) Literature for which the full text was not available or could not be downloaded.

The full text of studies that met the criteria was further reviewed by two independent researchers after an initial screening based on inclusion and exclusion criteria. If two researchers disagree on the assessment of the research literature reviewed, a third researcher is consulted and a decision made. A total of 16 eligible and relevant papers were finally included.

### 2.3 Outcome of assessment

This study selected decision-making accuracy, number of fixations, and duration of fixations as measurable indicators of perceptual-cognitive skills. The specific definition and measurement of these indicators are described below. The researchers of measured these indicators by recording participants' responses and visual characteristics when they watch the video clips identified by experts. Decision-making accuracy represents the frequency of producing in which an appropriate decision is made according to objective standards and task constraints. Decision-making performance are generally accepted as key indicators in domain of sports officials. Accuracy in included studies expressed as number of correct decisions or percentage of correct decisions. Moreover, number and duration of fixations as the indicators of visual search are measured by eye-tracking device.

### 2.4 Data extraction and analysis

For the eligible research literature, we extracted data on the name of the paper, type of campaign, subject profile, number of subjects, type of indicator, mean, and standard deviation. Furthermore, we referred to MacMahon's classification to divide sports officials into interactors (with high interaction and a large number of cues to process) such as soccer referee, monitors (with low interaction and a large number of cues to monitor) such as gymnastics judge, and reactors (with low interaction and a low number of cues to track) such as tennis line judge (MacMahon et al., 2014). For those studies that conducted more than two groups of experiments (e.g., expert group, intermediate group, and non-expert group), we mainly extracted and analyzed data from the highest and lowest levels of groups. When reviewing the full text and extracting data, we applied the modified Methodological Index for Non-Randomized Studies (MINORS) to systematically assess the reliability and validity of the full text (Slim et al., 2003; Supplementary Tables 2, 3). Meta-analyses were then performed using Review Manager 5.4 to quantitatively compare the effect size (SMD: standardized mean difference) between the two groups. According to Cohen's criteria for evaluating effect sizes: an effect size < 0.2 is a small effect, between 0.2 and 0.8 is a medium effect, and >0.8 is a large effect size (Rice and Harris, 2005). For each outcome, we calculated a weighted mean effect size and a 95% confidence interval (95% CI) for the mean to confirm whether the effect value was significantly different from zero. Higgins' *I*^2^ was also calculated to measure the degree of heterogeneity in effect sizes. If *I*^2^ ≤ 50%, a fixed effects model is selected; if *I*^2^ > 50%, a random-effects model is selected. In addition, publication bias was visually assessed by creating funnel plots (a simple scatter plot that reflects estimated effects of a single study with a given sample size) using Review Manager, and the risk of publication bias was further assessed by performing an Egger regression test on outcomes containing 10 or more studies using Stata 12.0. For all statistical tests, a two-sided *P* < 0.05 was considered statistically significant.

## 3 Result

**Figure 1:**
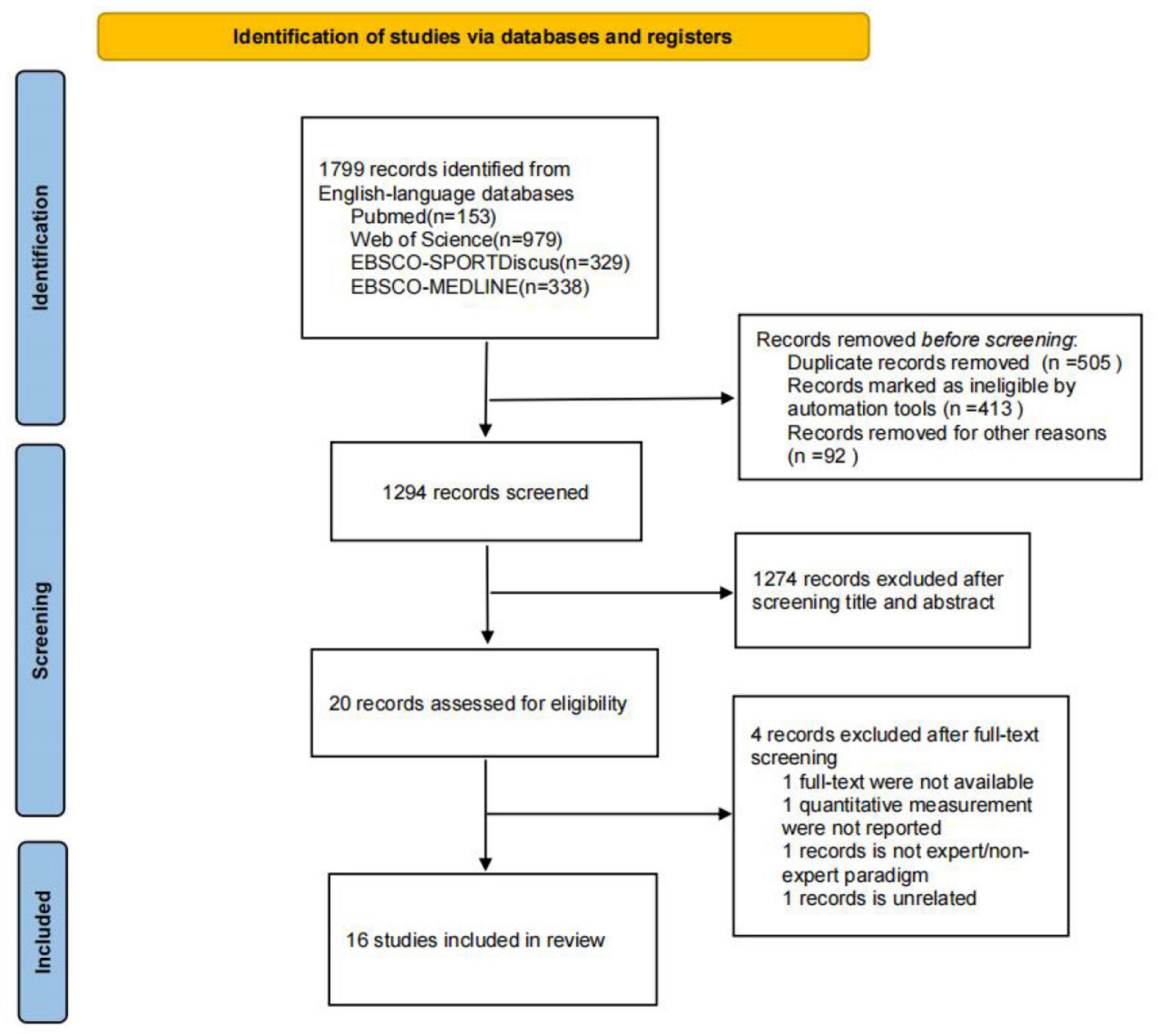
PRISMA flow diagram.

With a systematic search of several databases, a total of 1,799 relevant studies were retrieved. After careful reading and evaluation of the full text, a total of 16 studies were selected for inclusion (Figure 1). Among the included studies, football was the most commonly sport (6 studies), followed by gymnastics (2 studies) and rugby (2 studies). While basketball, fencing, ice hockey, cricket, Behind-the-plate baseball, and fast-pitch softball had only one study. More than half of the studies examined the decision-making accuracy (10 studies), number of fixations (10 studies), and duration of fixations (10 studies) of expert and non-expert sports officials (Table 1).

**Table 1:** Characteristics and effect sizes of included studies.

**References**	**Type of sports**	**Type of sport official**	**Number of participants**	**Indicator(s)**	**Value (mean ±SD)**
1. Mascarenhas et al. (2005); (English)	Rugby	Interactors	37 (exp14, non-exp23)	Accuracy	Exp: 54.3 ± 32.9
					Non-exp: 52.4 ± 26.3
2. Bard et al. (1980); (English)	Gymnastics	Monitors	7 (exp4, non-exp3)	Number of fixations	Compulsory
					Exp: 69.44 ± 22.28
					Non-exp: 94.65 ± 16.45
					Optional
					Exp: 94.65 ± 19.38
					Non-exp: 129.41 ± 52.33
3. Aghakhanpour et al. (2021); (English)	Fencing	Monitors	28 (exp14, non-exp14)	Accuracy	Exp: 43.21 ± 3.65
					Non-exp: 27.36 ± 5.82
				Number of fixations	Exp: 2.17 ± 0.47
					Non-exp: 3.03 ± 0.40
				Duration of fixations	Exp: 642.8 ± 159.7
					Non-exp: 522.7 ± 106.7
4. Kostrna and Tenenbaum (2022); (English)	Basketball	Interactors	56 (exp24, non-exp32)	Accuracy	Exp: 61.67 ± 7.42
					Non-exp: 31.35 ± 6.33
				Number of fixations	Exp: 3.44 ± 1.11
					Non-exp: 6.85 ± 1.55
				Duration of fixations	Exp: 397.59 ± 67.41
					Non-exp: 379.11 ± 99.80
5. Mack (2020); (English)	Gymnastics	Monitors	32 (exp14, non-exp18)	Duration of fixations	Original
					Exp: 0.457 ± 0.121
					Non-exp: 0.436 ± 0.109
					Stick-figure
					Exp: 0.506 ± 0.191
					Non-exp: 0.497 ± 0.169
				Number of fixations	Original
					Exp: 6.076 ± 1.034
					Non-exp: 6.282 ± 0.892
					Stick-figure
					Exp: 5.882 ± 1.166
					Non-exp: 5.816 ± 1.300
6. Hancock and Ste-Marie (2013); (English)	Ice hockey	Interactors	30 (exp15, non-exp15)	Number of fixations	Exp: 9.19 ± 2.27
					Non-exp: 9.18 ± 2.26
				Duration of fixations	Exp: 420.31 ± 112.02
					Non-exp: 459.29 ± 148.84
				Accuracy	Exp: 19.27 ± 1.44
					Non-exp: 17.73 ± 2.31
7. Ramachandran et al. (2021); (English)	Cricket	Interactors	31 (exp12, non-exp19)	Number of fixations	Exp: 5.0 ± 1.6
					Non-exp: 4.2 ± 1.7
				Duration of fixations	Exp: 972.91 ± 628.01
					Non-exp: 1,520.42 ± 663.90
8. Van Biemen et al. (2023); (English)	Football	Interactors	14 (exp5, non-exp9)	Accuracy	Exp: 87.8 ± 10.6
					Non-exp: 76.1 ± 14.7
				Duration of fixations	Exp: 400 ± 18
					Non-exp: 507 ± 12
9. Larkin et al. (2011); (English)	Football	Interactors	28 (exp15, non-exp13)	Accuracy	Exp: 23.7 ± 4.8
					Non-exp: 21.9 ± 4.2
10. Moore et al. (2019); (English)	Rugby	Interactors	18 (exp9, non-exp9)	Accuracy	Exp: 53.33 ± 14.14
					Non-exp: 38.89 ± 13.64
				Number of fixations	Exp: 1.64 ± 0.29
					Non-exp: 2.20 ± 0.19
11. Millslagle et al. (2013a); (English)	Behind-the-plate baseball	Interactors	8 (exp4, non-exp4)	Duration of fixations	Exp: 85.7% ± 14.3
					Non-exp: 49.8% ± 11.4
12. Spitz et al. (2018); (English)	Football	Interactors	43 (exp22, non-exp21)	Accuracy	Exp: 63.1 ± 9.8
					Non-exp: 55.4 ± 9.6
13. Millslagle et al. (2013b); (English)	Fast pitch softball	Interactors	8 (exp4, non-exp4)	Number of fixations	Exp: 2.06 ± 1.6
					Non-exp: 2.59 ± 1.8
14. Catteeuw et al. (2009); (English)	Football	Interactors	10 (exp5, non-exp5)	Accuracy	Exp: 83.5 ± 7.0
					Non-exp: 74.6 ± 4.8
15. Spitz et al. (2016); (English)	Football	Interactors	39 (exp20, non-exp19)	Accuracy	Open play technical
					Exp: 54.5 ± 19.2
					Non-exp: 49.5 ± 13.5
					Open play disciplinary
					Exp: 61.0 ± 17.0
					Non-exp: 45.3 ± 16.1
					Corner kick technical
					Exp: 69.5 ± 13.0
					Non-exp: 56.8 ± 10.5
					Corner kick disciplinary
					Exp: 82.5 ± 5.4
					Non-exp: 82.6 ± 7.4
				Number of fixations	Open play
					Exp: 16.9 ± 2.7
					Non-exp: 17.2 ± 2.6
					Corner kick
					Exp: 19.1 ± 2.2
					Non-exp: 19.6 ± 2.6
16. Van Biemen et al. (2022); (English)	Football	Interactors	12 (exp4, non-exp8)	Number of fixations	Exp: 1.3 ± 0.2
					Non-exp: 1.8 ± 0.3
				Duration of fixations	Exp: 1,158 ± 150
					Non-exp: 847 ± 240

### 3.1 Analysis of decision-making accuracy

**Figure 2:**
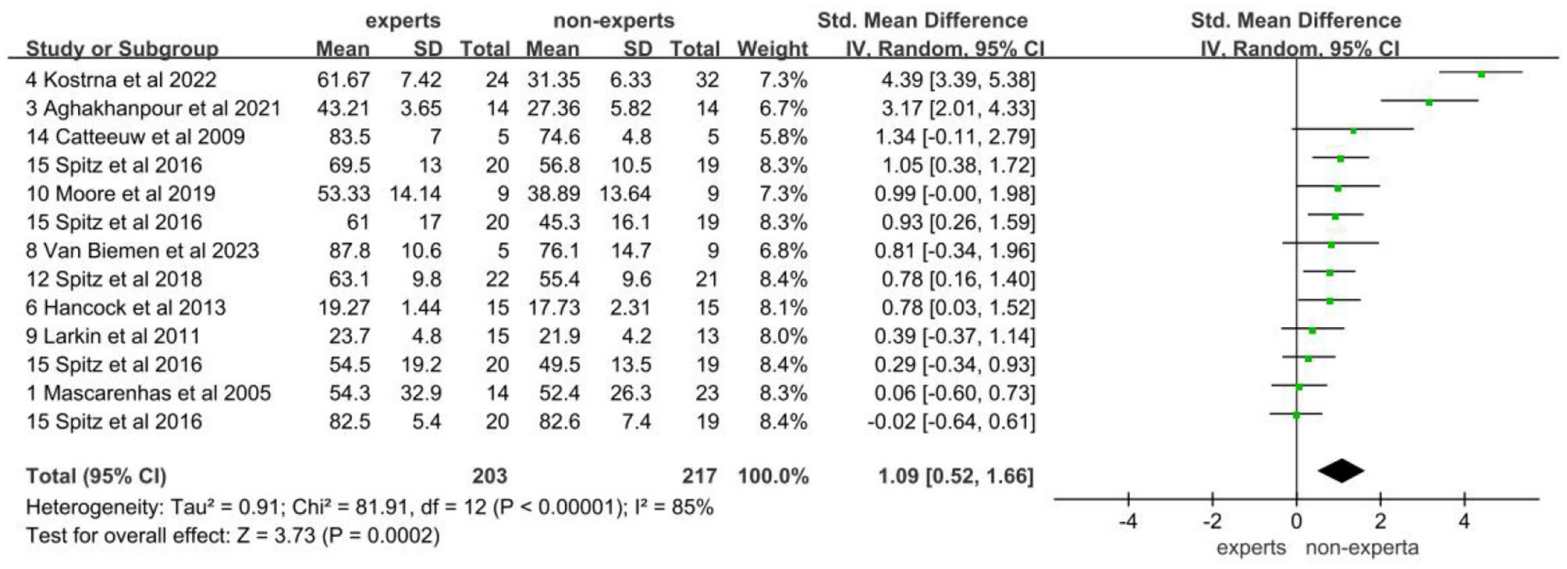
Forest plot of decision-making accuracy.

**Figure 3:**
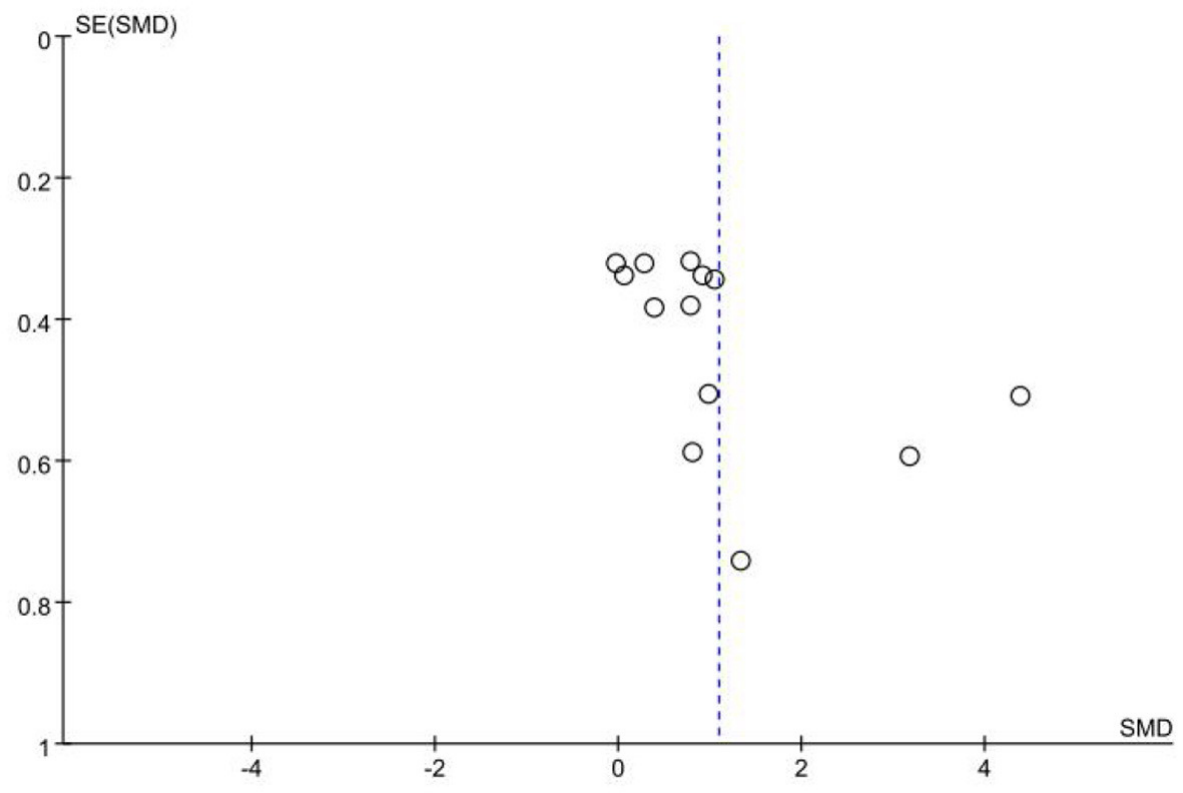
Funnel plot of decision-making accuracy.

The data of accuracy came from 10 studies involving 203 experts and 217 non-experts. After testing for heterogeneity, the heterogeneity of the studies was found to be high (*I*^2^ = 85%; *P* < 0.05), so a random effects model was chosen for the meta-analysis. The results showed a large effect size between the two groups, with an effect of 1.09 (95% CI: 0.52, 1.66; *P* < 0.05; Figure 2). It suggests that there is a significant difference in accuracy between expert and non-expert sports officials, with expert sports officials being significantly more accurate in their decision-making than non-expert sports officials. A visual assessment of the funnel plot revealed a possible publication bias (Figure 3). However, an Egger's test was performed and found that *P* = 0.124 was not statistically significant, implying that there was no publication bias.

### 3.2 Analysis of number of fixations

**Figure 4:**
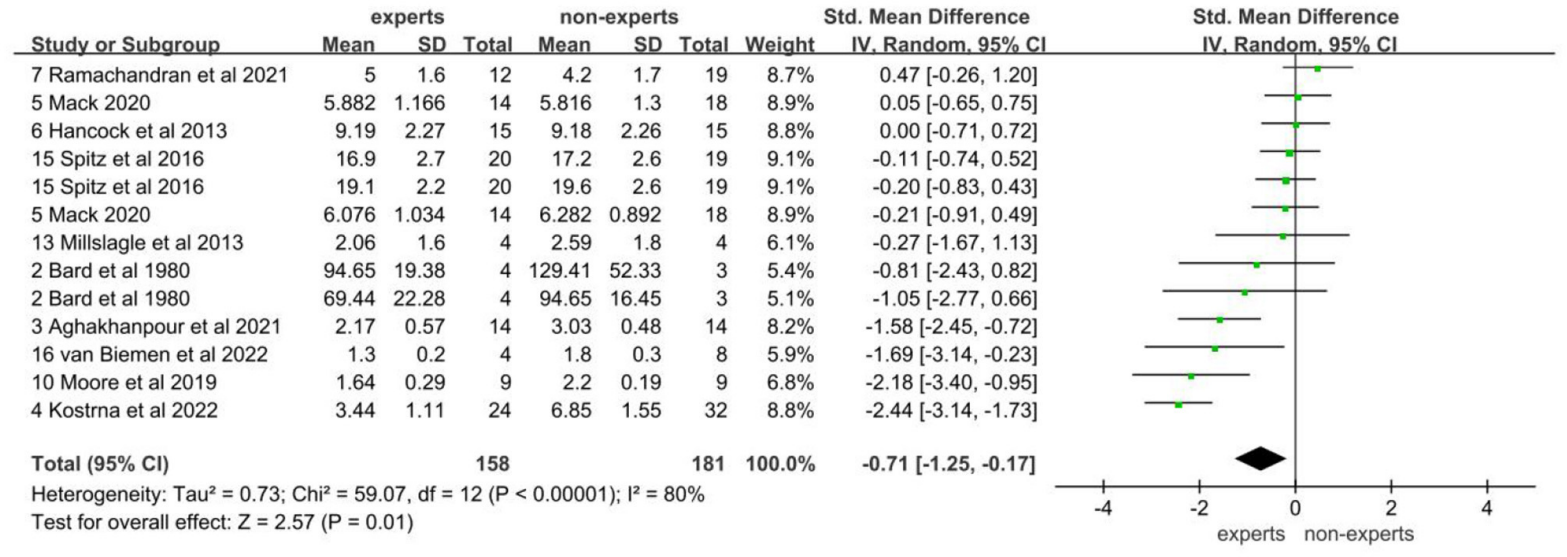
Forest plot of number of fixations.

**Figure 5:**
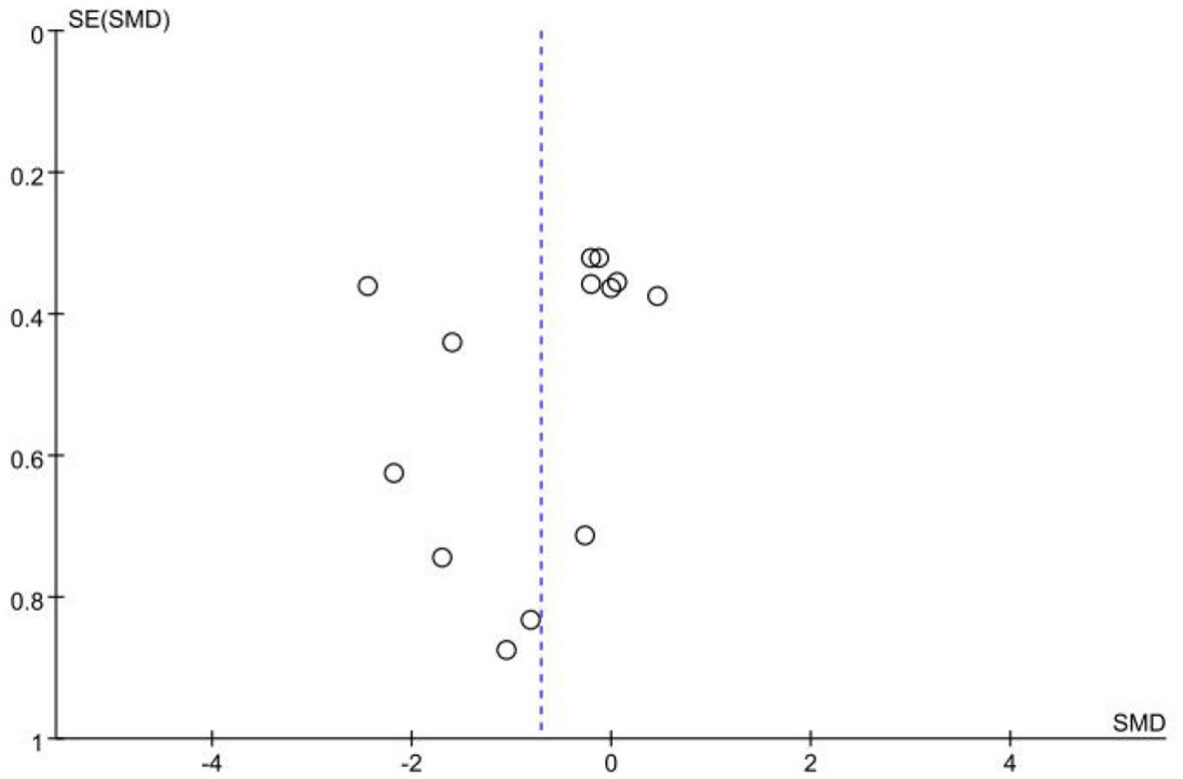
Funnel plot of number of fixations.

The data on the number of fixations came from 10 studies involving 158 experts and 181 non-experts. After testing for heterogeneity, it was found that there was a high degree of heterogeneity between studies (*I*^2^ = 80%; *P* < 0.05), so a random effects model was chosen for the meta-analysis. The results showed that there was a significant difference between expert and non-expert sports officials in the number of fixations, with expert sports officials having fewer fixations than non-expert sports officials. The effect size between the two groups was a medium effect, with an effect of −0.71 (95% CI: −1.25, −0.17; *P* < 0.05; Figure 4). After observing the funnel plot, it seems there might be a slight publication bias (Figure 5). However, the Egger's test indicates that a statistically insignificant *P*-value of 0.276 precludes the presence of publication bias.

### 3.3 Analysis of duration of fixations

**Figure 6:**
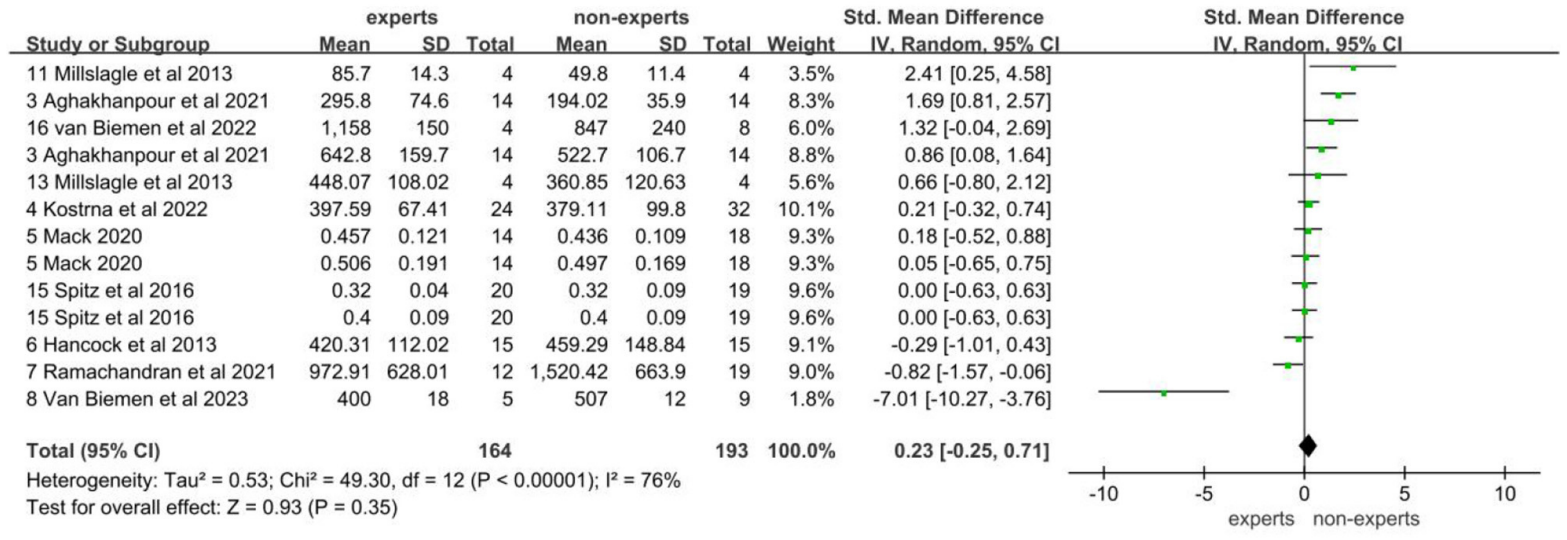
Forest plot of duration of fixations.

**Figure 7:**
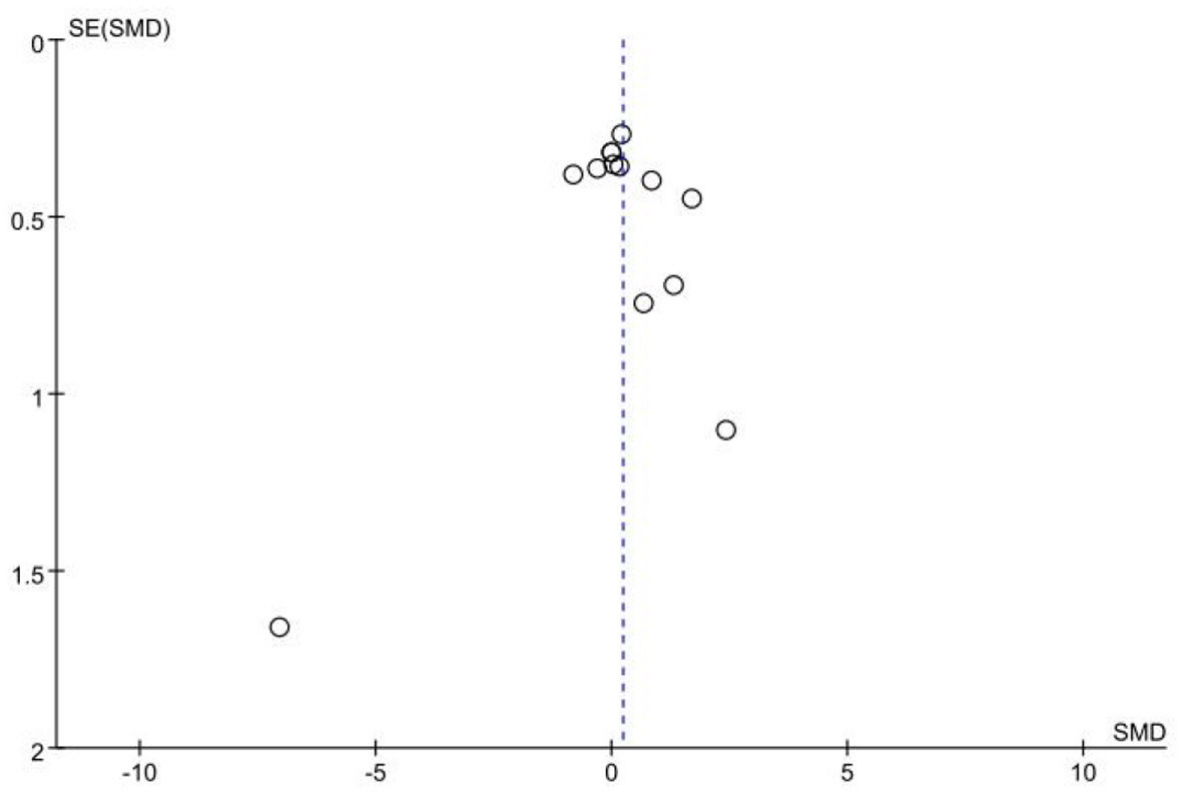
Funnel plot of duration of fixations.

The data on the duration of fixations came from 10 studies involving 164 experts and 193 non-experts. After testing for heterogeneity, it was found that there was a high degree of heterogeneity between studies (*I*^2^ = 76%; *P* < 0.05), so a random effects model was chosen for the meta-analysis. The results showed that there was no significant difference in duration of fixations between the expert and non-expert sports officials, with an effect size of 0.23 (95% CI: −0.25, 0.71; *P* = 0.35; Figure 6). After observing the funnel plot, it was felt that there was no publication bias (Figure 7). Egger's test was also performed and it was found that *P* = 0.956 was not statistically significant and therefore there was no publication bias.

### 3.4 Subgroup analysis

Sports officials were categorized into interactors, monitors and reactors according to MacMahon and Plessner (2007)'s classification, thus dividing the studies into three groups for analysis (Table 2). Among the studies included, there were 13 studies on interactors and three studies on monitors, with no studies on reactors. In the subgroup analyses we can find that there are some differences in the perceptual-cognitive skills of the various types of sports officials. In terms of accuracy, experts of interactors (SMD: 0.93; 95% CI: 0.39, 1.48) showed higher accuracy compared to non-experts. Furthermore, the difference in accuracy between experts and non-experts of monitors is greater than interactors. In terms of number of fixations, there are some differences between different types of sports officials. The experts of monitors showed fewer fixations compared to non-experts, with a medium effect size (SMD: −0.50; 95% CI: −0.91, −0.10). But there was no significant difference between the two groups of interactors (SMD: −0.75; 95% CI: −1.54, 0.03). In the terms of duration of fixations, there was no significant difference between experts and non-experts in both interactors (SMD: −0.01; 95% CI: −0.63, 0.61) and monitors (SMD: 0.66; 95% CI: −0.04, 1.35).

**Table 2:** Results of subgroup analyses.

**Variable**	**Type of sports officials**	**Number of studies**	**SMD (95% CI)**	***P*-value**	***I*^2^ (*P*)**
Decision-making accuracy	Interactors	9	0.93 [0.39, 1.48]	*P* < 0.05	83% (*P* < 0.05)
	Monitors	1	3.17 [2.01, 4.33]	*P* < 0.05	/
Number of fixations	Interactors	7	−0.75 [−1.54, 0.03]	*P* = 0.06	86% (*P* < 0.05)
	Monitors	3	−0.50 [−0.91, −0.10]	*P* < 0.05	58% (*P* = 0.05)
Duration of fixations	Interactors	8	−0.01 [−0.63, 0.61]	*P* = 0.96	76% (*P* < 0.05)
	Monitors	2	0.66 [−0.04, 1.35]	*P* = 0.06	70% (*P* < 0.05)

## 4 Discussion

### 4.1 Characteristics of difference in perceptual-cognitive skills

The purpose of this study is to systematically compare and assess the differences in perceptual-cognitive skills between expert and non-expert sports officials, and further explore the potential differences caused by different types of sports officials, in order to provide a more comprehensive understanding of the perceptual-cognitive skills of sports officials. To confirm the situation of differences between the expert and non-expert sports officials, we examined three main indicators: decision-making accuracy, number of fixations and duration of fixations. We conducted a systematic review and meta-analysis of the relevant literature to quantitatively synthesize the data from relevant studies to reveal the characteristics and reasons for the specific differences in perceptual-cognitive skills between expert and non-expert sports officials. The results of the study showed that there was a significant difference between expert and non-expert sports officials in terms of decision-making accuracy and number of fixations, but not in terms of duration of fixations.

Accuracy directly reflects the sports officials' competence and level of performance, and is a direct indicator for evaluating sports officials. The findings on accuracy are similar to the previous studies, and many researchers have confirmed that expert sports officials are more accurate than non-expert sports officials when making decisions (Spitz et al., 2018; Moore et al., 2019). We found that expert sports officials were significantly more accurate than non-expert sports officials and had a large effect sizes, suggesting that expert sports officials are able to use their expertise to make more accurate decisions based on objective criteria when officiating. Aghakhanpour et al. (2021) found that the number of correct decisions made by expert sports officials was significantly higher than novice sports officials, and argue that the time and experience that expert sports officials have gained over the years can help them to pick up a number of favorable cues by observing the movements of the athletes. Kostrna and Tenenbaum (2022) also argued that expert sports officials are able to develop better mental representations and more effective information processing strategies through training, resulting in more accurate decisions than novice sports officials. Taken together, accuracy seem to serve as an important indicator for measuring and distinguishing expert sports officials from non-expert sports officials.

The number of fixations reflects the sports official's proficiency in the perceptual task as well as the level of visual information collection and stability of visual control, with fewer fixations indicating that the sports official is more efficient at extracting information. The results demonstrate that the number of fixations made by expert sports officials is significantly lower than that of non-expert sports officials, implying that expert sports officials employ a more efficient visual search strategy. In the field of cognitive psychology, the strategy of visual search has long been an important tool to study the cognitive characteristics (Wilschut et al., 2014). In the study of athletes' visual search strategies, it was discovered that the number of fixations decreased as the level of athleticism increased. The reduction in the number of fixations by expert athletes is related to the perceptual-cognitive advantage they have developed over long periods of training and competition (Williams et al., 2012). This perceptual-cognitive advantage enabled expert athletes to conduct effective visual searches, thus focusing on the important and critical areas of information in the motor situation, which ultimately leads to fewer number of fixations. Similarly for sports officials, such a perceptual-cognitive advantage increases with the sports officials' level of officiating, resulting in fewer fixations and more efficient information extraction.

However, the result of duration of fixations in this study did not show significant differences and do not support the previously stated hypothesis that expert sports officials have longer duration of fixations. The duration of fixations reflects the efficiency of the sports official's attention allocation and information processing. Typically, longer duration of fixations result in greater extraction of information from the target, facilitating more accurate and rational decision-making. It has been suggested that the fixations of expert sports officials is characterized by longer duration, indicating that they are able to extract more relevant task information from each fixation (Mann et al., 2007). However, this conclusion is not always corroborated. As the perceptual-cognitive strategies of novice sports officials are not well-developed, it often takes longer duration of fixations to extract the information for making a decision (Mann et al., 2019). There is research even indicating that high sustained attention are not crucial skills and are not important for expert sports officials (Spitz et al., 2018). This also means that duration of fixations is not yet a valid indicator for distinguishing between expert and non-expert sports officials.

### 4.2 Reason of difference in perceptual-cognitive skills

MacMahon et al. (2014) had composed a decision-making model for sports officials and argued that perception is the first step in the whole decision-making process, followed by information categorization and integration, and finally the appropriate decision is made. The sports officials must continually perceive the information on the field of play (including the environment, athletes, and even colleagues), and use specific visual search strategies to collect relevant information while ignoring irrelevant information that may disrupt decision-making (Helsen and Bultynck, 2004). It is indisputable that the sport official's perceptual-cognitive skills, as a crucial element of early information collection in the decision-making process, are critical to make accurate and efficient decisions.

The results of the study show that there are significant differences in accuracy and the number of fixations between expert and non-expert sports officials, indicating the variance in their perceptual-cognitive skills. The perceptual-cognitive advantage possessed by expert sports officials can assist them in making more accurate decisions and optimizing their visual search strategy when officiating to some degree. In recent years, researchers have extensively utilized eye-tracking device to investigate the perceptual-cognitive skills of sports officials, trying to determine the primary factors causing differences in such skills. After conducting a systematic review of relevant literature, we propose four potential factors that may influence the perceptual-cognitive skills of sports officials.

Firstly, we argue that task anticipation behavior of sports officials leads to differences in information perception. Expert sports officials can exhibit more superior perceptual-cognitive skills than non-expert sports officials due to their abundant reserves of professional expertise and extensive experience. Having accumulated such experience, expert sports officials are able to generate specific knowledge and perceptual skills in the areas they are familiar with, which facilitate the gathering and processing of information in their officiating process. This ability not only helps them to make better decisions, but also to make efficient and accurate anticipations (Ericsson and Kintsch, 1995). Anticipation is the act of expecting or predicting what may happen in the future based on prior knowledge, experience, or intuition. It has been shown that anticipation allows experts to expend fewer resources and use more effective visual search strategies to scan relevant environmental information (Mann et al., 2007). Expert athletes can alleviate the time constraints of the task by making faster and more accurate decisions than non-experts through their outstanding predictions. Likewise, expert sports officials also can perceive and process information on the field of play earlier with the help of anticipation (Williams et al., 2002). As a result, sports officials can direct their vision earlier, concentrating on zones where potential fouls may occur, ultimately decreasing the number of fixations and improving decision-making accuracy. In contrast, non-expert sports officials displayed limited knowledge and experience, leading to extensive information gathering through various visual search behavior. This in turn resulted in more fixations and lower efficiency in collecting information, ultimately lowering decision-making accuracy.

Secondly, we argue that different methods of memory retrieval lead to differences in information processing. When officiating, sports officials sometimes draw on their own memories of previous events to generate responses that help them to better perceive and process information. Previous research has shown that experts appear to be able to retain experience and knowledge from training and competition in long-term memory. They can quickly extract information when necessary to perform actions with speed and accuracy (Ericsson and Chase, 1982). Plessner and Haar (2006) in their finding of OSDMM (Official's Specific Decision-Making Model) also mentioned that the process of decision-making by sport official begins with the official perceiving the stimulus. Next, the stimulus is encoded, interpreted, and categorized, assisted by long-term memory. Then, the sports officials integrates the perceived stimulus and information with their retrieved memories and any additional information into decision-making. Furthermore, as performers become more expert, they have been shown to be able to use their working memory more effectively (Ericsson, 2008). As expert sports officials will usually have more experience, they will develop more refined information retrieval strategies and processing methods. Expert sports officials are able to extract relevant information from long-term memory more efficiently when confronted with comparable situations, directing their visual attention and making accurate decisions. Thus, expert sports officials demonstrate fewer fixations and higher decision-making accuracy. In contrast, non-expert sports officials have less officiating experience, limited content stored in long-term memory, and rely on more random visual behavior to collect information when faced with unfamiliar and complex tasks.

Thirdly, we argue that information reduction strategy lead to differences in sources of information. Some scholars have attempted to explain differences in perceptual-cognitive skills between experts and non-experts using the information-reduction hypothesis (Haider and Frensch, 1999). This hypothesis suggests that experts are able to optimize the amount of information processed through selective gaze behavior, ignoring task-irrelevant information and actively focusing on task-relevant information. In most sports, sports officials are often required to make accurate and reasonable decisions in a relatively short period of time, but complex tasks and time constraints can make sports officials' decision-making behavior extremely difficult. In such time-constrained tasks, experts are better able to distinguish between relevant and irrelevant sources of information and focus their attention on the most important sources of information (Brams et al., 2019). Expert sports officials have learned over many years of officiating experience to use targeted visual search strategies to ignore irrelevant cues in the task, selectively focusing on task-relevant information to reduce and optimize the amount of information they have to process. The lower number of fixations demonstrated by experts is because they reduce the information-processing load and minimize the need for sensory input to create a coherent perception of the task situation (Aghakhanpour et al., 2021).

Fourthly, it has been proposed that differences in perceptual-cognitive skills are linked to the particular sport and task nature. The perceptual-cognitive demands on sports officials vary from sport to sport. In order to reduce the impact of item differences, we used MacMahon's classification of sports officials to further compare and analyze the perceptual-cognitive skills of expert and non-expert sports officials (MacMahon and Plessner, 2007). MacMahon categorized sports officials into interactors, monitors, and reactors based on two main aspects: the amount of interaction with athletes and movement demands, and in the number of cues being observed. Interactors with high interaction and physical movement demands and often a large number of cues to process, such as soccer and basketball referees. Monitors with low to medium interaction and physical demands, but often a medium to large number of cues to monitor, such as volleyball and gymnastics judges. Reactors with low interaction and movement demands and a low to medium number of cues to track, such as tennis line judges. Among these three categories of sports officials, reactors make judgment based on objective facts without any involvement of perceptual-cognitive skills in the process of adjudication. As a result, there is limited academic research on the perceptual-cognitive skills of reactor, and we will not discuss this type of sports official in our study. Our findings confirm that the type of sports officials does not affect accuracy and duration of fixations. However, accuracy can be used as a useful parameter to differentiate between expert and non-expert decision-making performance, while duration of fixation does not. The results concerning number of fixations indicate that interactors and monitors exhibit different perceptual-cognitive skills due to the task's particular nature. Interactors need to pay attention to a large number of different targets and cues, in addition to frequent movements and interactions during officiating. This also renders the visual search task of such sports officials more intricate, with greater uncertainty in decision-making. In conclusion, differences in sports do result in differences in the perceptual-cognitive skills of sports officials. Therefore, the specifics of the sport need to be taken into account when evaluating the perceptual-cognitive skills of sports officials.

Furthermore, differences in perceptual-cognitive skills of sports officials may also be influenced by other factors. Such as the type of stimulus and the presentation of decision clips. Different types of stimuli and presentation may cause sports officials to perceive and extract different information when watching the materials. This study focuses on the potential impact of the types of sports officials, but future studies are needed to explore the effects of other factors on the perceptual-cognitive skills of expert and non-expert sports officials.

## 5 Conclusion

This study employed meta-analysis to comprehensively analyze and compare the perceptual-cognitive skills of expert and non-expert sports officials. The results of the study show that accuracy can be an important indicator of perceptual-cognitive skills in distinguishing expert and non-expert sports officials. Number of fixations can be important factors of perceptual-cognitive skills in distinguishing between experts and non-experts in sport monitors, and the perceptual-cognitive skills of experts are significantly better than those of non-experts in this type of sports official. As MacMahon states, perceptual-cognitive skills are crucial in sports officials and are being emphasized by a growing number of scholars. The training and development of sports officials can try to start from the perceptual-cognitive skills to improve the efficiency of sports officials in information collection and processing, thus helping them to make timely and accurate decisions more efficiently.

## 6 Limitations and future directions

This study has the following three limitations. Firstly, the sample size included in the study was limited, which to some extent may have made the results statistically insignificant. Secondly, the inclusion of multiple sports in this study and the different definitions of “expert” and “non-expert” sports officials in different studies resulted in a high degree of heterogeneity between the included studies. Although we used MacMahon's classification of sports officials to group the studies, we could not completely eliminate the heterogeneity between studies. Thirdly, most of the studies included in this review were tested in a laboratory setting, and the data collected may have differed from reality. Future research into the perceptual-cognitive skills of sports officials can be considered in the following areas. Firstly, different eye movement indicators were selected as outcome variables to further explore the characteristics of sports officials' gaze behavior. Secondly, a specific sport was used as the object of the study to explore the differences in perceptual-cognitive skills of different sports officials in the same sport. Thirdly, other potential factors that may influence the perceptual-cognitive skills of expert sports officials, such as the testing environment, measurement tools and presentation of clips, can be further explored.

## Data availability statement

The original contributions presented in the study are included in the article/Supplementary material, further inquiries can be directed to the corresponding author.

## Author contributions

YW: Conceptualization, Formal analysis, Methodology, Project administration, Writing – original draft, Writing – review & editing. ZY: Formal analysis, Investigation, Writing – review & editing. RW: Formal analysis, Investigation, Writing – review & editing. HZ: Methodology, Supervision, Writing – review & editing. QZ: Conceptualization, Funding acquisition, Project administration, Writing – review & editing, Writing – original draft.
