# Supplementary material for: A comparison of perceptual-cognitive skills in expert and non-expert sports officials: a systematic review and meta-analysis
Source: Front Psychol. 2024 Jun 21;15:1380281. doi: 10.3389/fpsyg.2024.1380281 (PMC11224550; doi:10.3389/fpsyg.2024.1380281)
Supplement: Supplementary file 1 [file Data_Sheet_1.PDF]

Supplementary material

Table S1. Search strategy for the seven databases

Database	Step	Search strategy	Number of articles
Pubmed	#1	"anticipation"[Title] OR "prediction"[Title] OR "decision-making"[Title] OR "expertise"[Title] OR "cue use"[Title] OR "information processing"[Title] OR "cognitive characteristics"[Title] OR "visual search"[Title] OR "visual attention"[Title] OR "visual fixation"[Title] OR "eye movement"[Title] OR "eye-tracking"[Title] OR "occlusion"[Title] OR "expert"[Title] OR "non-expert"[Title] OR "amateur"[Title] OR "novice"[Title] OR "elite"[Title] OR (("perceptual"[Title] OR "cognitive"[Title]) AND "skill"[Title])	232,681
	#2	"referee"[Title] OR "judge"[Title] OR "judgement"[Title] OR "umpire"[Title] OR "official"[Title] OR "officiate"[Title]	6,581
	#3	-2022/12/31	-
	#4	Language:English	-
	#5	#1 AND #2 AND #3 AND #4	153
Web of Science	#1	TI=anticipation OR prediction OR decision-making OR expertise OR cue use OR information processing OR cognitive characteristics OR visual search OR visual attention OR visual fixation OR eye movement OR eye-tracking OR occlusion OR expert OR non-expert OR amateur OR novice OR elite OR ((perceptual OR cognitive) AND skill)	338,380
	#2	TI=referee OR judge OR judgement OR umpire OR official OR officiate	23,867
	#3	-2022/12/31	-
	#4	Language:English	-
	#5	#1 AND #2 AND #3 AND #4	979
EBSCO-SPORTDiscus	#1	((((TI+perceptual)+OR+(TI+cognitive))+AND+(TI+skill))+OR+(TI+anticipation+OR+TI+prediction+OR+TI+decision-making+OR+TI+expertise+OR+TI+cue+use+OR+TI+information+processing+OR+TI+cognitive+characteristics+OR+TI+visual+search+OR+TI+visual+attention+OR+TI+visual+fixation+OR+TI+eye+movement+OR+TI+eye-tracking)+OR+(TI+occlusion+OR+TI+expert+OR+TI+non-expert+OR+TI+amateur+OR+TI+novice+OR+TI+elite))	32,097
	#2	(TI+referee+OR+TI+judge+OR+TI+judgement+OR+TI+umpire+OR+TI+official+OR+TI+officiate)	10,507
	#3	-2022/12/31	-
	#4	Language:English	-
	#5	#1 AND #2 AND #3 AND #4	329
EBSCO-MEDLINE complete	#1	((((TI+perceptual)+OR+(TI+cognitive))+AND+(TI+skill))+OR+(TI+anticipation+OR+TI+prediction+OR+TI+decision-making+OR+TI+expertise+OR+TI+cue+use+OR+TI+information+processing+OR+TI+cognitive+characteristics+OR+TI+visual+search+OR+TI+visual+attention+OR+TI+visual+fixation+OR+TI+eye+movement+OR+TI+eye-tracking)+OR+(TI+occlusion+OR+TI+expert+OR+TI+non-expert+OR+TI+amateur+OR+TI+novice+OR+TI+elite))	265,922

#2	(TI+referee+OR+TI+judge+OR+TI+judgement+OR+TI+umpire+OR+TI+official+OR+TI+officiate)	10,466
#3	-2022/12/31	-
#4	Language:English	-
#5	#1 AND #2 AND #3 AND #4	338

Table S2. Modified MINORS system for evaluating the risk of bias of the included

Item	Score
1. The study's aim is stated	0=unreported, 1=reported but insufficient, 2=reported and sufficient
2. Participants at different levels included	0=unreported, 1=reported but insufficient, 2=reported and sufficient
3. Appropriate method for collecting data	0=unreported, 1=reported but insufficient, 2=reported and sufficient
4. Endpoint appropriate for the study's aim	0=unreported, 1=reported but insufficient, 2=reported and sufficient
5. Unbiased evaluation of endpoints	0=unreported, 1=reported but insufficient, 2=reported and sufficient
6. A comparable control group	0=unreported, 1=reported but insufficient, 2=reported and sufficient
7. Baseline equivalence of groups	0=unreported, 1=reported but insufficient, 2=reported and sufficient
8. Statistical analyses adapted to the study's design	0=unreported, 1=reported but insufficient, 2=reported and sufficient

Table S3. Scores of the included studies according to MINORS

Item	Mascarenhas et al	Bard et al	Aghakhanpour et al	Kostrna et al	Mack	Hancock et al	Ramachandran et al	Van Biemen et al
1. The study's aim is stated	2	2	2	2	2	2	2	2
2. Participants at different levels included	2	2	2	2	2	2	2	2
3. Appropriate method for collecting data	2	2	2	2	2	2	2	2
4. Endpoint appropriate for the study's aim	2	2	2	1	2	2	1	2
5. Unbiased evaluation of endpoints	2	2	2	2	2	2	2	2
6. A comparable control group	2	2	2	2	1	2	2	2
7. Baseline equivalence of groups	2	2	2	2	2	2	2	2
8. Statistical analyses adapted to the study's design	1	1	2	2	1	1	1	1
Total	15	15	16	15	14	15	14	15

Item	Larkin et al	Moore et al	Millslagle et al	Spitz et al	Millslagle et al	Catteeuw et al	Spitz et al	van Biemen et al
1. The study's aim is stated	2	2	2	2	2	2	2	2
2. Participants at different levels included	2	2	2	2	2	2	2	2
3. Appropriate method for collecting data	1	2	2	2	2	2	2	2
4. Endpoint appropriate for the study's aim	2	2	2	2	2	2	2	2
5. Unbiased evaluation of endpoints	2	2	2	2	2	2	2	2
6. A comparable control group	2	2	2	2	2	2	2	2
7. Baseline equivalence of groups	2	2	2	2	2	2	2	2
8. Statistical analyses adapted to the study's design	1	2	1	2	1	1	2	1
Total	14	16	15	16	15	15	16	15
